# Plasmon-enhanced optoacoustic transducer with Ecoflex thin film for broadband ultrasound generation using overdriven pulsed laser diode

**DOI:** 10.1117/1.JBO.28.12.125005

**Published:** 2023-12-22

**Authors:** Hamin Na, Jaehyeok Park, Ki-Hun Jeong

**Affiliations:** a Korea Advanced Institute of Science and Technology (KAIST), Department of Bio and Brain Engineering, Daejeon, Republic of Korea; b Korea Advanced Institute of Science and Technology (KAIST), KAIST Institute for Health Science and Technology (KIHST), Daejeon, Republic of Korea

**Keywords:** optoacoustic transducer, plasmonic photothermal effect, broadband ultrasound, laser modulation, acoustic atomization

## Abstract

**Significance:**

Ultrasonic transducers facilitate noninvasive biomedical imaging and therapeutic applications. Optoacoustic generation using nanoplasmonic structures provides a technical solution for highly efficient broadband ultrasonic transducer. However, bulky and high-cost nanosecond lasers as conventional excitation sources hinder a compact configuration of transducer.

**Aim:**

Here, we report a plasmon-enhanced optoacoustic transducer (PEAT) for broadband ultrasound generation, featuring an overdriven pulsed laser diode (LD) and an Ecoflex thin film. The PEAT module consists of an LD, a collimating lens, a focusing lens, and an Ecoflex-coated 3D nanoplasmonic substrate (NPS).

**Approach:**

The LD is overdriven above its nominal current and precisely modulated to achieve nanosecond pulsed beam with high optical peak power. The focused laser beam is injected on the NPS with high-density electromagnetic hotspots, which allows for the efficient plasmonic photothermal effect. The thermal expansion of Ecoflex finally generates broadband ultrasound.

**Results:**

The overdriven pulsed LD achieves a maximum optical peak power of 40 W, exceeding the average optical power of 3 W. The 22  μm thick Ecoflex-coated NPS exhibits an eightfold optoacoustic enhancement with a fractional −6  dB bandwidth higher than 160% and a peak frequency of 2.5 MHz. In addition, the optoacoustic amplitude is precisely controlled by the optical peak power or the laser pulse width. The PEAT-integrated microfluidic chip clearly demonstrates acoustic atomization by generating aerosol droplets at the air–liquid interface.

**Conclusions:**

Plasmon-enhanced optoacoustic generation using PEAT can provide an approach for compact and on-demand biomedical applications, such as ultrasound imaging and lab-on-a-chip technologies.

## 1 Introduction

Ultrasound allows biocompatible and contactless bioimaging and therapeutic applications such as ultrasound imaging,^1^ high-intensity focused ultrasound treatment,^2^ and ultrasonic drug delivery.^3^ Conventional ultrasonic transducers often utilize piezoelectric (PZT) materials, which induce ultrasonic waves through mechanical vibration.^4^ However, the resonant behavior of PZT materials results in a narrow bandwidth^5^ and low transmission efficiency due to a large impedance mismatch between PZT ($Z>30\times 10^{6}\;\mathrm{kg}\cdot m^{-2}\cdot s^{-1}$) and biological tissues ($Z∼1.5\times 10^{6}\;\mathrm{kg}\cdot m^{-2}\cdot s^{-1}$).^6^ In addition, they require complicate steps of micropackaging such as precise mechanical dicing^7^ and multiple electrical connections.^8^ Micromachined ultrasonic transducers (MUTs) such as piezoelectric MUTs^9^ and capacitive MUTs^10^^,^^11^ have emerged as an alternative solution for technical limitations of conventional PZT transducers. Wafer-scale microfabrication of highly integrated MUTs allows high-density transducer arrays with controllable resonant frequency and broad bandwidth through variation of diaphragm sizes.^12^ However, they still exhibit technical bottlenecks such as multiple electrical connections,^13^ undesirable crosstalk,^14^^,^^15^ and complicated microfabrication.^16^

Optoacoustic transducers with nanosecond pulsed light and micro/nanoscale optical absorbers have introduced an approach for broadband ultrasound generation. The pulsed light excitation induces light-to-heat conversion in the optical absorbers, resulting in thermal expansion and subsequent generation of broadband ultrasound in the surrounding medium.^17^^,^^18^ High optoacoustic pressure is achieved by combining optical absorbers with high light absorption and thermal expanding polymer matrices with a high coefficient of thermal expansion (CTE).^19^^–^^21^ Polydimethylsiloxane (PDMS) serves as a prevalent thermal expanding layer due to the high CTE of $266.5\;\mu m\cdot m^{-1}\cdot {{}^{\circ}C}^{-1}$, compared with water and other polymers.^22^ Optical absorbers can be further improved with carbon-based materials such as carbon-nanotubes,^23^^–^^25^ candle soot nanoparticles,^26^ and carbon nanofibers,^27^ or metallic surfaces using Al,^28^ Cr,^29^^,^^30^ and Au.^31^ Recently, plasmonic nanostructures with localized surface plasmon resonance have been employed in optoacoustic transducers, thanks to the plasmonic photothermal effect as well as ultrathin thickness.^32^^–^^34^ Electromagnetic hotspots between two-dimensional or three-dimensional nanostructures significantly enhance the optoacoustic amplitude.^35^^,^^36^ For example, three-dimensional Ag nanostructures significantly enhance the optoacoustic amplitude by more than 20 times compared to a polymeric substrate without plasmonic structures.^19^ However, the common utilization of bulky Q-switched Nd:YAG lasers with nanosecond pulse widths (PWs; <10  ns) impedes the system integration into a compact optoacoustic module, despite the progress in optical absorbers. Optoacoustic generation utilizing semiconductor light sources, such as light-emitting diodes^37^^–^^39^ and laser diodes (LDs),^40^^–^^42^ have been limited to optoacoustic imaging applications and have not led to substantial progress in transducer development.

**Fig. 1:**
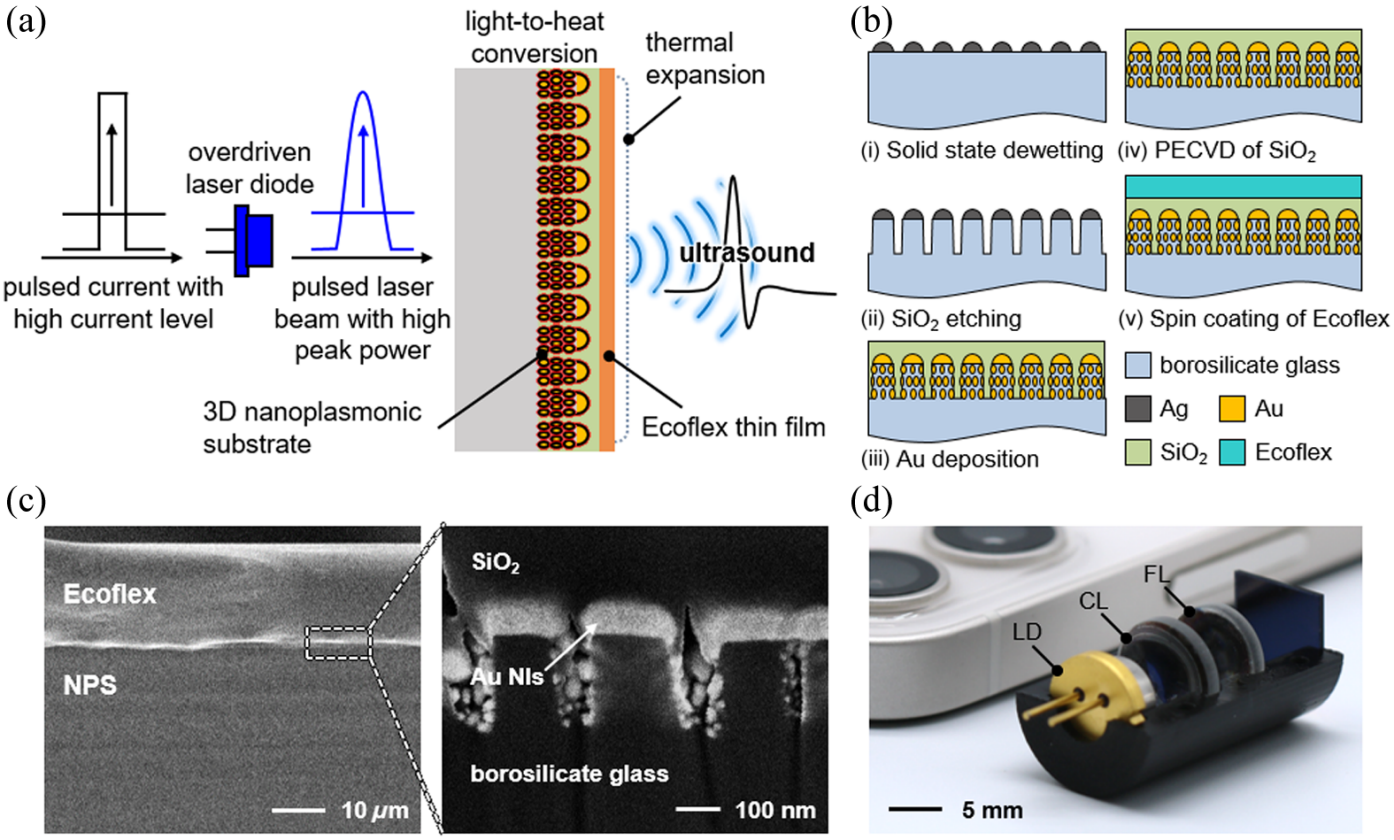
PEAT using focused pulsed laser beam. (a) A schematic illustration of PEAT including Ecoflex-coated NPS. (b) Nanofabrication procedures of Ecoflex-coated NPS using solid state dewetting of Ag thin film, reactive ion etching, Au thin film evaporation, PECVD of ${\mathrm{SiO}}_{2}$, and spin-coating of the thin Ecoflex film. (c) Cross-sectional SEM images of the Ecoflex-coated NPS. (d) Fully packaged PEAT module consisting of an LD, a CL, an FL, and an Ecoflex-coated NPS.

Here, we report compact plasmon-enhanced optoacoustic transducer (PEAT) for broadband ultrasound therapy and imaging applications by utilizing an overdriven pulsed LD. Figure 1(a) illustrates the working principle of PEAT including a thin polymer film-coated nanoplasmonic substrate (NPS) and a single LD. The LD is overdriven above the nominal current level by applying high pulsed current, resulting in pulsed laser beam with high optical peak power. The focused laser beam is injected on the NPS with highly dense electromagnetic hotspots, which allows efficient photothermal conversion due to high light absorption. The thermoelastic expansion of polymer thin film finally generates broadband optoacoustic waves.

## 2 Materials and Methods

### 2.1 Nanofabrication of Ecoflex-Coated NPS

The nanofabrication procedure of Ecoflex-coated NPS is shown in Fig. 1(b). Glass nanopillar arrays (GNAs) were fabricated using thermal evaporation and annealing of Ag thin film and reactive ion etching of the glass wafer. Nanogap-rich NPS was fabricated using thermal evaporation of Au thin film on the GNAs, and the plasma-enhanced chemical vapor deposition (PECVD) of ${\mathrm{SiO}}_{2}$. The Ecoflex-coated NPS was finally prepared by spin-coating and curing the Ecoflex prepolymer. Figure 1(c) shows the cross-sectional scanning electron microscopy (SEM) image of the Ecoflex-coated NPS. The fabricated Ecoflex-coated NPS exhibits a high optical absorption, primarily due to the abundant nanogaps between Au nanoislands, as shown in Fig. S1 in the Supplementary Material. The fully packaged PEAT module consists of an LD; an aspheric lens for collimation, i.e., collimating lens (CL); a plano-convex lens for focusing, i.e., focusing lens (FL); and an Ecoflex-coated NPS. The chip size of Ecoflex-coated NPS (9  mm×9  mm) was adjusted to the module size of the LD. The physical dimension of the PEAT module shows 14  mm×25  mm×14  mm, as shown in Fig. 1(d).

### 2.2 Plasmon-Enhanced Optoacoustic Generation System Using Overdriven LD

**Fig. 2:**
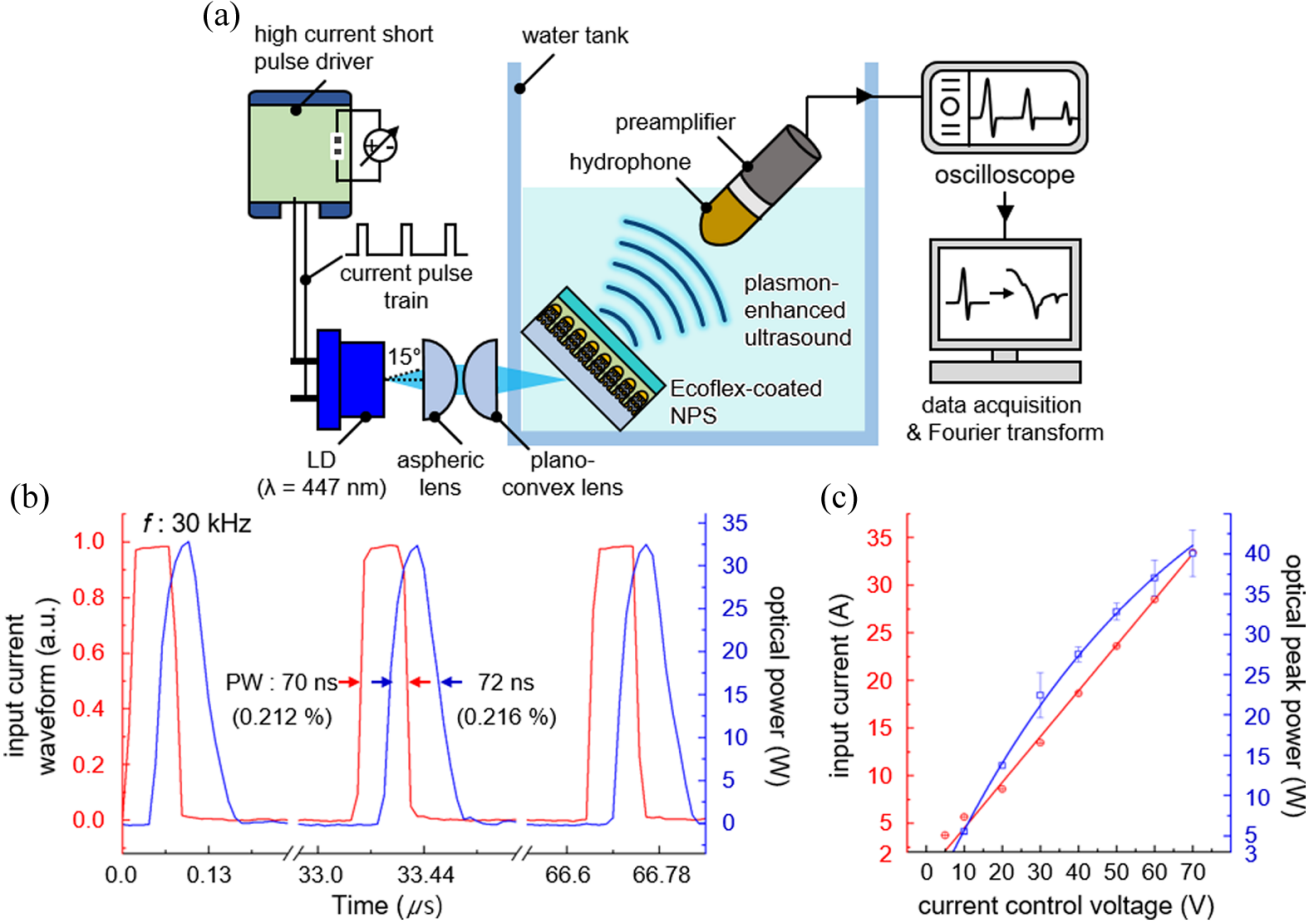
LD-based plasmon-enhanced optoacoustic generation system. (a) Experimental setup for light modulation and underwater optoacoustic measurement. (b) Time-lapsed input current waveform and optical peak power of the LD. (c) Input current and optical peak power of the overdriven LD depending on the current control voltage.

A LD-based plasmon-enhanced optoacoustic generation system is shown in Fig. 2. Figure 2(a) shows the experimental setup for underwater optoacoustic measurement using overdriven LD. A current pulse train of a nanosecond PW is generated by a high current short pulse driver based on the preconfigured square waveform. The input current level is adjusted with the current control voltage to exceed the nominal current level of the LD. A laser beam is initially collimated with the aspheric lens, and then precisely focused on the back side of the NPS with the plano-convex lens. The NPS induces thermal expansion of the thin Ecoflex film and leads to ultrasound generation. The ultrasonic waves are measured with a hydrophone positioned at 45 deg to avoid baseline distortion resulted from high optical power, as shown in Fig. S2 in the Supplementary Material. Note that the hydrophone detects all optoacoustic waves at the incident angles below 20 deg, exhibiting the maximum sensitivity for optoacoustic waves at the normal incidence. The optoacoustic signal is finally amplified with a 20-dB preamplifier and acquired with a LabVIEW interface via an oscilloscope with an input impedance of 50 Ω.^43^ The time-domain waveforms were converted into the frequencies using Fourier transform in MATLAB. The optical image of experimental setup is shown in Fig. S3 in the Supplementary Material. Figure 2(b) shows the time-lapsed waveforms of input current and laser beam. The optical pulse train is highly correlated with the current pulse train, operating at a frequency (f) of 30 kHz and a PW of 70 ns. Note that turn-on delay times of 40 and 2 ns longer optical PW result from the throughput delay of the high current short pulse driver^44^ and the inductance on an LD packaging,^41^ respectively. Figure 2(c) shows the input current and the optical peak power of the overdriven LD depending on the current control voltage. The input current linearly increases with the current control voltage, whereas the optical peak power tends to level off due to the carrier leakage.^45^ The optical peak power reaches 40 W at the input current of 33 A, which is approximately an order of magnitude higher than the average optical power of 3 W at the nominal current of 2 A.

## 3 Results and Discussion

### 3.1 Plasmon-Enhanced Optoacoustic Generation via PEAT Depending on Thin Ecoflex Film

**Fig. 3:**
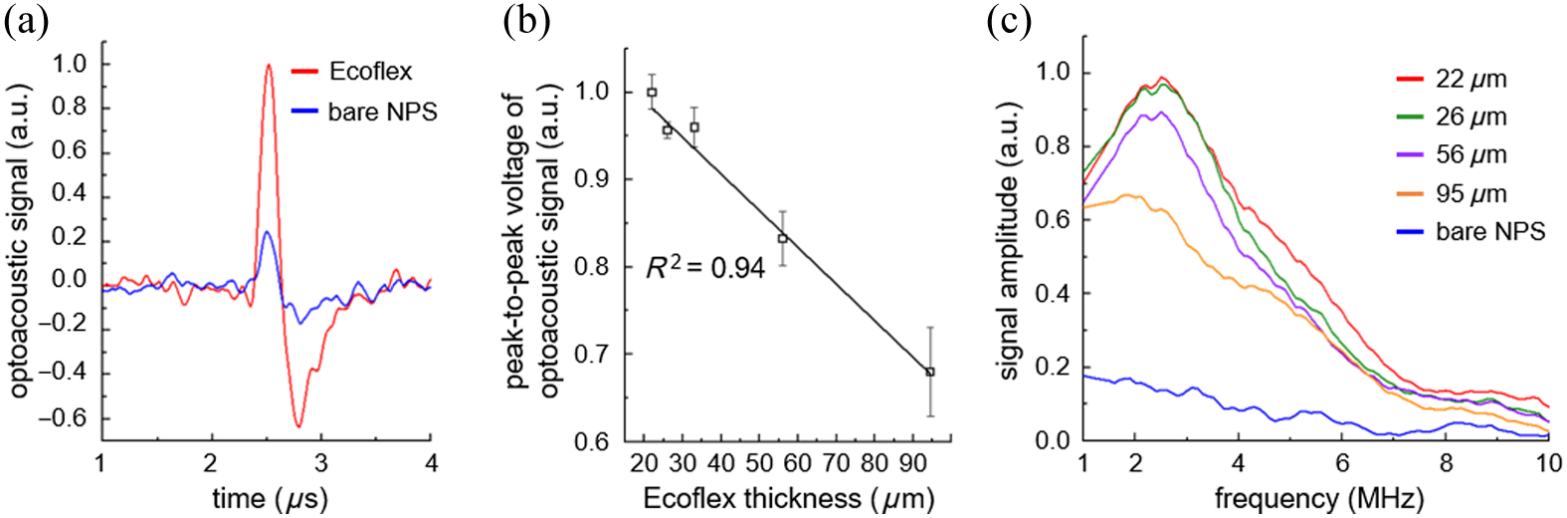
Plasmon-enhanced optoacoustic generation via PEAT depending on thin Ecoflex film. (a) Optoacoustic signals from 25  μm thick Ecoflex-coated NPS and a bare NPS. (b) Peak-to-peak voltage of optoacoustic signals and (c) frequency responses from Ecoflex-coated NPSs with various thicknesses of Ecoflex.

Plasmon-enhanced optoacoustic generation has been experimentally demonstrated with PEAT, as shown in Fig. 3. Figure 3(a) shows optoacoustic signals from 25  μm thick Ecoflex-coated NPS and a bare NPS under illumination with a laser beam with the optical peak power of 33 W and PW of 70 ns at a current control voltage of 50 $V_{\mathrm{DC}}$. The measured optoacoustic signals exhibit distinct positive and negative peaks of compressive and tensile waves due to thermoelastic expansion and contraction of the thin polymer film. The Ecoflex-coated NPS significantly enhances the optoacoustic amplitude by a factor of 4 due to the large thermal expansion of Ecoflex. Note that Ecoflex has a CTE of $284.2\;\mu m\cdot m^{-1}\cdot {{}^{\circ}C}^{-1}$ and thus the optoacoustic amplitude is enhanced by a ratio of CTE of Ecoflex to PDMS, as shown in Table S1 in the Supplementary Material.^22^ Figure 3(b) shows peak-to-peak voltages of optoacoustic signals from Ecoflex-coated NPSs depending on the thickness of Ecoflex. The thicknesses of Ecoflex were controlled with different speed of spin-coating, ranging from 1000 to 6000 rpm. Thinner Ecoflex films enhance optoacoustic signals by enabling rapid heat transfer from the NPS, converting highly confined thermal energy directly into optoacoustic waves without thermal diffusion.^46^ Figure 3(c) shows the frequency responses of the corresponding optoacoustic signals. The measured ultrasonic frequencies from the Ecoflex-coated NPS exhibit an eightfold amplitude increase at 2.5 MHz compared with the bare NPS. The experimental results clearly show that the peak frequency is inversely proportional to the square root of the thickness of thermal expanding layer.^47^ For instance, the 22  μm thick Ecoflex film increases broadband and high-frequency components with a fractional −6  dB bandwidth higher than 160%, and a peak frequency of 2.5 MHz. Note that the fractional −6  dB bandwidth of conventional PZT transducer is around 45%.^6^ In contrast, the 95  μm thick Ecoflex film shows relatively lower frequencies around 1.8 MHz with the fractional −6  dB bandwidth of 230%.

### 3.2 Plasmon-Enhanced Optoacoustic System via PEAT Depending on Laser Modulation

**Fig. 4:**
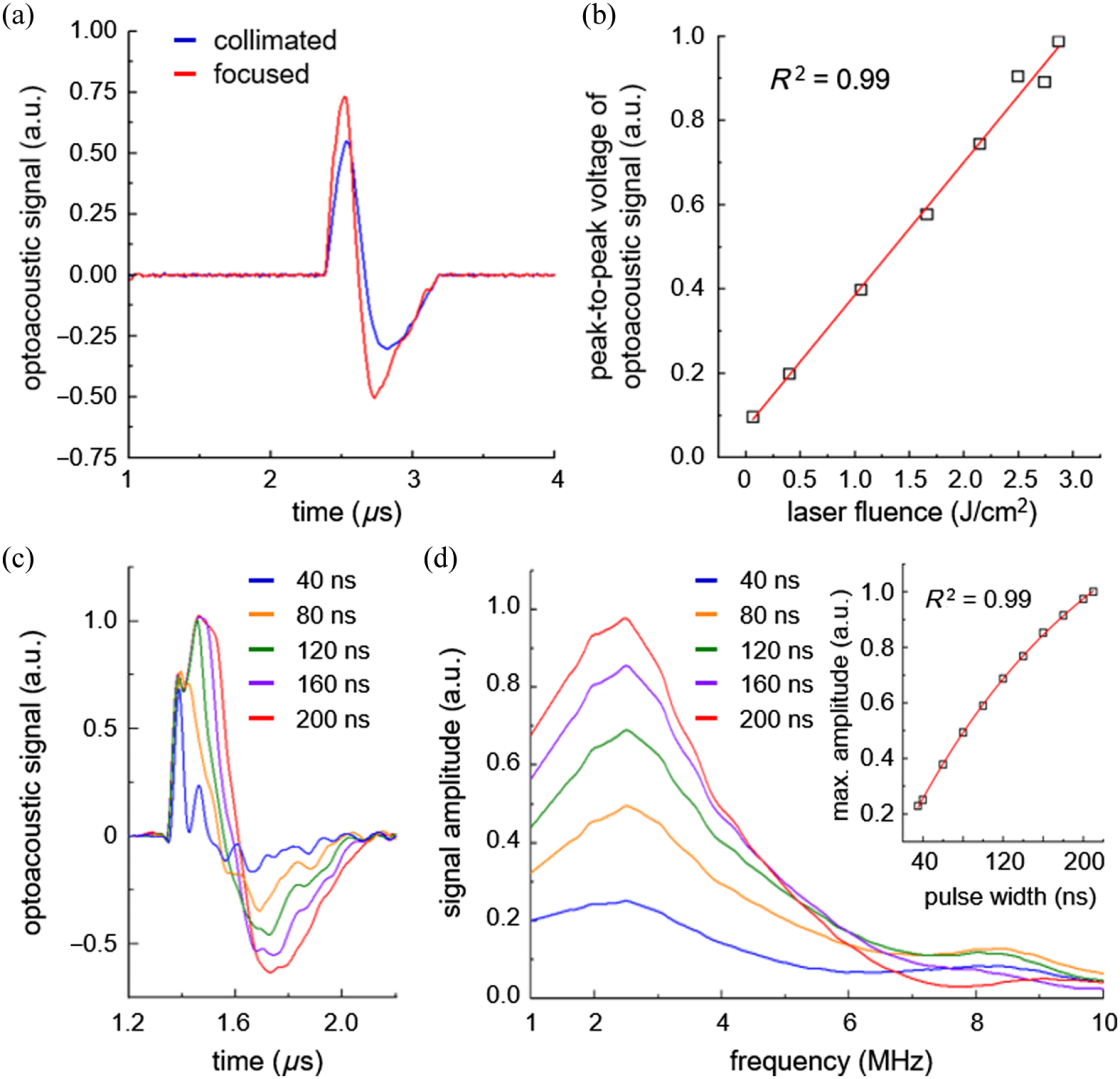
Plasmon-enhanced optoacoustic generation via PEAT depending on laser modulation. (a) Optoacoustic signals from the Ecoflex-coated NPS induced by collimated and focused laser beams. (b) Peak-to-peak voltages of optoacoustic signals depending on the laser fluence. (c) Optoacoustic signals from the PEAT depending on the PW. (d) Frequency responses of the optoacoustic signals and maximum amplitude depending on the PW (inset).

Laser modulation dependent plasmon-enhanced optoacoustic generation has been experimentally demonstrated with PEAT, as shown in Fig. 4. Figure 4(a) shows the optoacoustic signals using collimated and focused laser beams with an optical peak power of 33 W and a PW of 160 ns. A large illumination area of collimated beam [full width at half maximum (FWHM) of beam diameter: 800  μm] generates ultrasound in a wide area beyond the focal spot of focused beam (FWHM of beam diameter: 16  μm). The focused laser beam increases the optoacoustic amplitude by 1.7 times compared with the collimated laser beam due to intense laser fluence confined within the focused spot. Figure 4(b) shows peak-to-peak voltages of optoacoustic signals depending on the laser fluence of the focused beam. The laser fluence was modulated by controlling the current control voltage within the range of 5 to 70 V. The peak-to-peak voltage of optoacoustic signals linearly increase with the laser fluence, which demonstrates the strong linearity between the optoacoustic pressure and the laser fluence.^48^ Note that the optoacoustic amplitude is proportional to the temporal derivative of the corresponding optical pulse, as shown in Fig. S4 in the Supplementary Material.^49^ Figure 4(c) shows the optoacoustic signals depending on the PW of focused laser beam. The optoacoustic signals become broaden and increase in the amplitude with the PW. As the laser PW increases, the positive peak saturates at the PW of 120 ns, as shown in Fig. S5 in the Supplementary Material, whereas the negative peak rises due to the enhanced Gruneisen coefficient caused by the temperature increase in the Ecoflex film.^50^^,^^51^ The optoacoustic amplitude at the PW of 200 ns corresponds to a peak-to-peak pressure around 0.6 kPa at the 2 mm distance from the Ecoflex-coated NPS. The Nd:YAG nanosecond laser generates optoacoustic amplitude enhanced by two orders of magnitude compared to the overdriven LD. However, the intense output pulse energy from the laser results in damage and delamination of Ecoflex thin film, as shown in Fig. S6 in the Supplementary Material. The optoacoustic amplitude can be further improved by employing structural modifications such as composite thermal expansion layer^52^ or high absorbance optical cavity.^29^ Figure 4(d) shows the frequency responses of the corresponding optoacoustic signals. The maximum amplitude exhibits a quadratic growth with the laser PW and finally levels off due to thermal energy saturation.^53^ As a result, the optimal thickness of Ecoflex and the laser modulation allow the precise control of the output optoacoustic waves, particularly the optoacoustic amplitude and the peak frequency.

### 3.3 Experimental Demonstration of Acoustic Atomization via PEAT

**Fig. 5:**
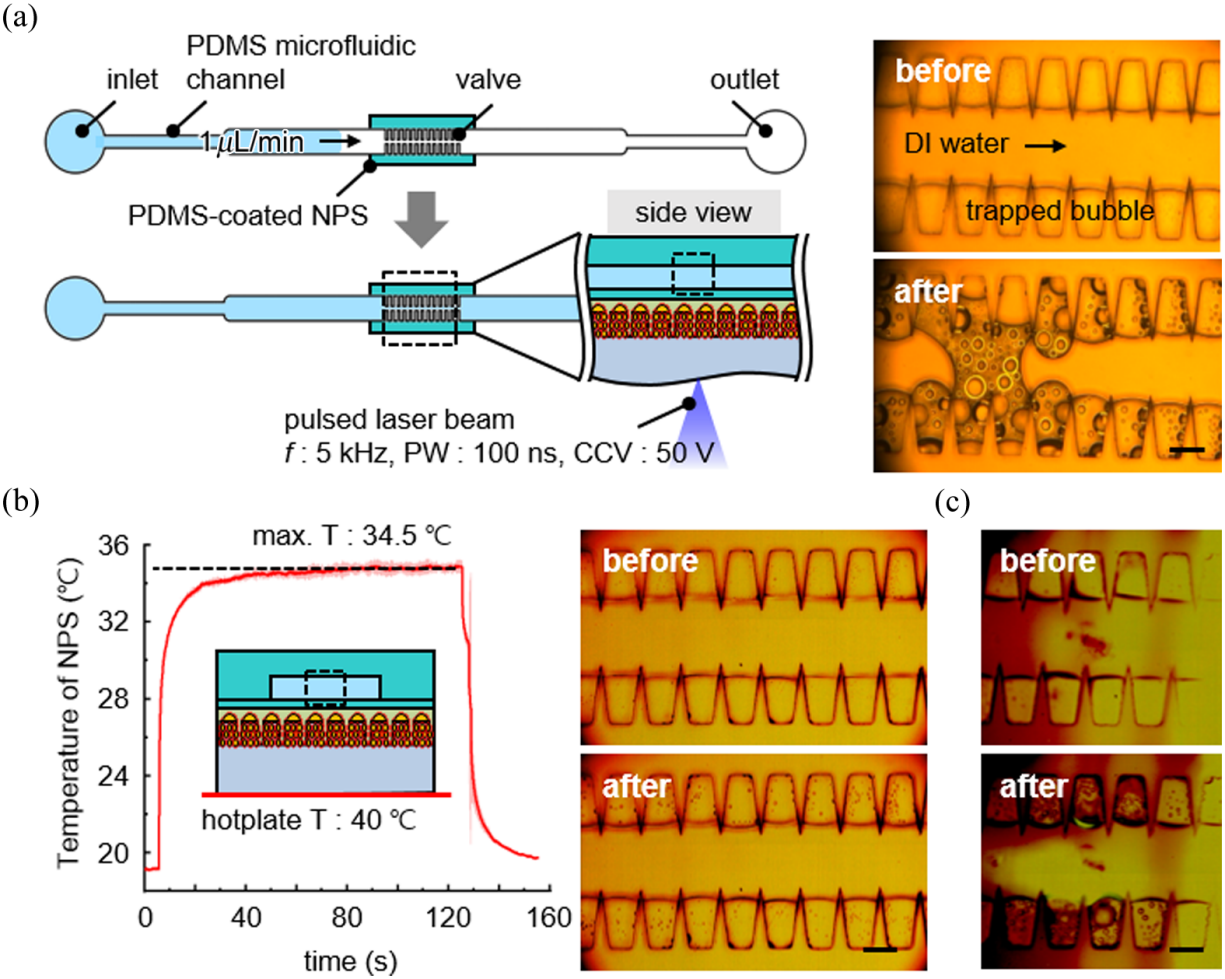
Experimental demonstration of acoustic atomization via PEAT. (a) Experimental setup for acoustic atomization (left) and optical images before and after pulsed laser irradiation for 30 s (right). (b) Experimental setup for verifying the influence of photothermal effect on acoustic atomization (left), and optical images before and after heating the microfluidic chip (right). The light-to-heat conversion of NPS results in negligible expansion of trapped bubbles without forming an air slug in the microchannel. (c) Optical images before and after the irradiation of pulsed laser beam on the PDMS microfluidic chip bonded to bare glass wafer. All scale bars represent 100  μm.

The acoustic atomization of liquid inside a microfluidic chip has been experimentally demonstrated with PEAT, as shown in Fig. 5. Figure 5(a) shows the experimental setup for acoustic atomization in PDMS microfluidic channel and the optical images before and after the laser irradiation for 30 s. A PDMS-coated NPS is utilized instead of the Ecoflex-coated NPS for ensuring tight adhesion between the microfluidic chip and PEAT by oxygen plasma treatment. The PDMS microfluidic chip features microgroove structures for trapping air bubbles, whereas the middle microchannel is filled with injected fluid such as deionized (DI) water. The irradiation of pulsed laser beam with a frequency of 5 kHz and a PW of 100 ns generates the movement of trapped microbubbles and aerosol microdroplets at the air–liquid interface. The coalescence and expansion of moving microbubbles blocks the flow of DI water by forming an air slug at the middle of microchannel. Figure 5(b) shows the influence of photothermally generated heat on the acoustic atomization. The surface temperature NPS was measured using an infrared thermographic camera. The NPS reaches the maximum temperature of 34.5°C after 2 min of pulsed laser beam irradiation. The microfluidic chip bonded to the PDMS-coated NPS was placed on a hotplate set at 40°C, exceeding the maximum temperature of 34.5°C. The mild heat of 40°C results in negligible bubble expansion without the formation of an air slug. Figure 5(c) shows the influence of pulsed laser beam on the acoustic atomization. The pulsed laser beam is directed onto the PDMS microfluidic chip bonded to a bare glass wafer with perfect optical transmittance. The minimal impact of pulsed laser beam ensures that high-frequency optoacoustic waves induce the atomization of liquid. As a result, the acoustically driven microfluidic atomization facilitates various microfluidic functions such as the development of microbubble-based microvalves, without additional pumping units.

## 4 Conclusions

We have successfully demonstrated the PEAT for broadband ultrasound generation. The PEAT features the LD, the aspheric lens, the plano-convex lens, and the Ecoflex-coated NPS. The overdriven pulsed LD achieves the maximum optical peak power of 40 W, which is ∼10-fold higher than the average optical power of 3 W. The experimental results clearly show that the optimal Ecoflex thickness and laser modulation allow for precise control of the optoacoustic waves, particularly the amplitude and the peak frequency. Finally, optoacoustic waves within the PEAT-integrated microfluidic chip induce atomization at the air–liquid interface and create an air slug in the end. This optoacoustic transducer can provide a platform of ultrasound generation for on-demand and compact biomedical applications, including ultrasound imaging and lab-on-a-chip technologies.

## Data Availability

The datasets that support the findings of this article are available from the corresponding author upon reasonable request.

## References

[1] Patricio Rodrigues E. et al., “Development of a 2-D array ultrasonic transducer for 3-D imaging of objects immersed in water,” Sensors 21(10), 3501 (2021). SNSRES 0746-9462 10.3390/s21103501 34069762 PMC8157294

[2] Duc N. M. Keserci B., “Emerging clinical applications of high-intensity focused ultrasound,” Diagn. Interv. Radiol. 25(5), 398 (2019). 10.5152/dir.2019.18556 31287428 PMC6727814

[3] Pitt W. G. Husseini G. Staples B. J., “Ultrasonic drug delivery—a general review,” Expert Opin. Drug Deliv. 1(1), 37 (2004). 10.1517/17425247.1.1.37 16296719 PMC1361256

[4] An Y. K. Kim M. K. Sohn H., “Piezoelectric transducers for assessing and monitoring civil infrastructures,” Sens. Technol. Civil Infrastruct. 1, 86–120 (2014). 10.1533/9780857099136.86

[5] Kim J. Y. et al., “Design of piezoelectric acoustic transducers for underwater applications,” Sensors 23(4), 1821 (2023). SNSRES 0746-9462 10.3390/s23041821 36850418 PMC9966007

[6] Li J. et al., “Recent advancements in ultrasound transducer: from material strategies to biomedical applications,” BME Front. 2022, 9764501 (2022). 10.34133/2022/9764501 37850168 PMC10521713

[7] Chan J. et al., “Photoacoustic imaging with capacitive micromachined ultrasound transducers: principles and developments,” Sensors 19(16), 3617 (2019). SNSRES 0746-9462 10.3390/s19163617 31434241 PMC6720758

[8] Qiu Y. et al., “Piezoelectric micromachined ultrasound transducer (PMUT) arrays for integrated sensing, actuation and imaging,” Sensors 15(4), 8020–8041 (2015). SNSRES 0746-9462 10.3390/s150408020 25855038 PMC4431219

[9] Griggio F. et al., “Micromachined diaphragm transducers for miniaturised ultrasound arrays,” in IEEE Int. Ultrason. Symp. (2012). 10.1109/ULTSYM.2012.0503

[10] Manwar R. Chowdhury S., “Experimental analysis of bisbenzocyclobutene bonded capacitive micromachined ultrasonic transducers,” Sensors 16(7), 959 (2016). SNSRES 0746-9462 10.3390/s16070959 27347955 PMC4970013

[11] Oralkan Ö. et al., “Capacitive micromachined ultrasonic transducers: next-generation arrays for acoustic imaging?” IEEE Trans. Ultrason. Ferroelectr. Freq. Control 49(11), 1596–1610 (2002). ITUCER 0885-3010 10.1109/TUFFC.2002.1049742 12484483

[12] Hajati A. et al., “Three-dimensional micro electromechanical system piezoelectric ultrasound transducer,” Appl. Phys. Lett. 101(25), 253101 (2012). APPLAB 0003-6951 10.1063/1.4772469

[13] Ritter T. A. et al., “A 30-MHz piezo-composite ultrasound array for medical imaging applications,” IEEE Trans. Ultrason. Ferroelectr. Freq. Control 49(2), 217–230 (2002). ITUCER 0885-3010 10.1109/58.985706 11885679

[14] Mo J. H. et al., “Crosstalk reduction with a micromachined diaphragm structure for integrated ultrasound transducer arrays,” IEEE Trans. Ultrason. Ferroelectr. Freq. Control 39(1), 48–53 (1992). ITUCER 0885-3010 10.1109/58.166810 18263118

[15] Yang Y. et al., “An ultra-high element density pMUT array with low crosstalk for 3-D medical imaging,” Sensors 13(8), 9624–9634 (2013). SNSRES 0746-9462 10.3390/s130809624 23896705 PMC3812571

[16] Bang S. et al., “Engineering-aligned 3D neural circuit in microfluidic device,” Adv. Healthc. Mater. 5(1), 159–166 (2016). 10.1002/adhm.201500397 26332914

[17] Attia A. B. E. et al., “A review of clinical photoacoustic imaging: current and future trends,” Photoacoustics 16(July), 100144 (2019). 10.1016/j.pacs.2019.100144 31871888 PMC6911900

[18] Chen S. L., “Review of laser-generated ultrasound transmitters and their applications to all-optical ultrasound transducers and imaging,” Appl. Sci. 7(1), 25 (2016). 10.3390/app7010025

[19] Park S. G. et al., “Plasmon enhanced photoacoustic generation from volumetric electromagnetic hotspots,” Nanoscale 8(2), 757–761 (2016). NANOHL 2040-3364 10.1039/C5NR05505A 26659557

[r20] Chen J. et al., “Deep-subwavelength control of acoustic waves in an ultra-compact metasurface lens,” Nat. Commun. 9(1), 4920 (2018). NCAOBW 2041-1723 10.1038/s41467-018-07315-6 30467347 PMC6250707

[21] Chen Y. et al., “Fully planar laser-generated focused ultrasound transmitter,” Sens. Actuators A Phys. 349, 113929 (2023). 10.1016/j.sna.2022.113929

[22] Lee T.-I. I. Kim M. S. Kim T.-S. S., “Contact-free thermal expansion measurement of very soft elastomers using digital image correlation,” Polym. Test. 51, 181–189 (2016). POTEDZ 0142-9418 10.1016/j.polymertesting.2016.03.014

[23] Baac H. W. et al., “Carbon-nanotube optoacoustic lens for focused ultrasound generation and high-precision targeted therapy,” Sci. Rep. 2(1), 989 (2012). SRCEC3 2045-2322 10.1038/srep00989 23251775 PMC3524551

[r24] Chen Z. et al., “Multilayered carbon nanotube yarn based optoacoustic transducer with high energy conversion efficiency for ultrasound application,” Nano Energy 46, 314–321 (2018). 10.1016/j.nanoen.2018.02.006

[25] Wang L. et al., “Ultrawide-bandwidth high-resolution all-optical intravascular ultrasound using miniaturized photoacoustic transducer,” Sci. Adv. 9(23), eadg8600 (2023). STAMCV 1468-6996 10.1126/sciadv.adg8600 37294755 PMC10256152

[26] Chang W. Y. et al., “Candle soot nanoparticles-polydimethylsiloxane composites for laser ultrasound transducers,” Appl. Phys. Lett. 107(16), 161903 (2015). APPLAB 0003-6951 10.1063/1.4934587

[27] Hsieh B. Y. et al., “A laser ultrasound transducer using carbon nanofibers-polydimethylsiloxane composite thin film,” Appl. Phys. Lett. 106(2), 21902 (2015). APPLAB 0003-6951 10.1063/1.4905659

[28] Zhang F. Krishnaswamy S. Lilley C. M., “Bulk-wave and guided-wave photoacoustic evaluation of the mechanical properties of aluminum/silicon nitride double-layer thin films,” Ultrasonics 45(1–4), 66–76 (2006). ULTRA3 0041-624X 10.1016/j.ultras.2006.06.064 16899268

[29] Zheng C. et al., “High-efficient photoacoustic generation with an ultrathin metallic multilayer broadband absorber,” Opt. Express 29(6), 8490 (2021). OPEXFF 1094-4087 10.1364/OE.420138 33820295

[30] Lee T. et al., “Highly efficient photoacoustic conversion by facilitated heat transfer in ultrathin metal film sandwiched by polymer layers,” Adv. Opt. Mater. 5(2), 1600421 (2017).2195-1071 10.1002/adom.201600421

[31] Yue S. et al., “Gold-implanted plasmonic quartz plate as a launch pad for laser-driven photoacoustic microfluidic pumps,” Proc. Natl. Acad. Sci. U. S. A. 116(14), 6580–6585 (2019). 10.1073/pnas.1818911116 30872482 PMC6452654

[32] Kang M. et al., “Bioplasmonic alloyed nanoislands using dewetting of bilayer thin films,” ACS Appl. Mater. Interfaces 9(42), 37154–37159 (2017). AAMICK 1944-8244 10.1021/acsami.7b10715 28949500

[r33] Kang B.-H. H. et al., “Ultrafast and real-time nanoplasmonic on-chip polymerase chain reaction for rapid and quantitative molecular diagnostics,” ACS Nano 15(6), 10194–10202 (2021). ANCAC3 1936-0851 10.1021/acsnano.1c02154 34008961

[34] Yu E.-S. et al., “Highly efficient on-chip photothermal cell lysis for nucleic acid extraction using localized plasmonic heating of strongly absorbing Au nanoislands,” ACS Appl. Mater. Interfaces 15, 34323–34331 (2023). AAMICK 1944-8244 10.1021/acsami.3c01856 37435756 PMC10375432

[35] Shi Y. Cui D. Zhang Z., “Quantitative study of the nonlinearly enhanced photoacoustic/photothermal effect by strong LSPR-coupled nanoassemblies,” Nanomaterials 10(10), 1942 (2020). 10.3390/nano10101942 33003437 PMC7601439

[36] Shen T. W. et al., “Plasmonic gold nanomaterials as photoacoustic signal resonant enhancers for cysteine detection,” Nanomaterials 11(8), 1887 (2021). 10.3390/nano11081887 34443721 PMC8401226

[37] Beard P. C. Allen T. J., “High power visible light emitting diodes as pulsed excitation sources for biomedical photoacoustics,” Biomed. Opt. Express 7(4), 1260–1270 (2016). BOEICL 2156-7085 10.1364/BOE.7.001260 27446652 PMC4929638

[r38] Hariri A. et al., “The characterization of an economic and portable LED-based photoacoustic imaging system to facilitate molecular imaging,” Photoacoustics 9, 10–20 (2018). 10.1016/j.pacs.2017.11.001 29234601 PMC5723278

[39] Agano T. et al., “Comparative experiments of photoacoustic system using laser light source and LED array light source,” Proc. SPIE 9323, 93233X (2015). PSISDG 0277-786X 10.1117/12.2077357

[40] Gallego D. Sanchez M. Lamela H., “High current short pulse driver using a high power diode laser for optoacoustic biomedical imaging techniques,” Opt. Express 30(25), 44954–44966 (2022). OPEXFF 1094-4087 10.1364/OE.476159 36522908

[r41] Stylogiannis A. et al., “Continuous wave laser diodes enable fast optoacoustic imaging,” Photoacoustics 9, 31–38 (2018). 10.1016/j.pacs.2017.12.002 29387537 PMC5772504

[42] Zeng L. et al., “Portable optical-resolution photoacoustic microscopy with a pulsed laser diode excitation,” Appl. Phys. Lett. 102(5), 53704 (2013). APPLAB 0003-6951 10.1063/1.4791566

[43] Na H. et al., “Plasmon-induced photoacoustic transducer for non-invasive skin tightening,” Proc. SPIE 12434, 124340E (2023). PSISDG 0277-786X 10.1117/12.2649566

[44] “Pulse laser diode driver module, PCO-7121 | Directed energy,” https://directedenergy.com/product/pco-7121/ (accessed 6 July 2023).

[45] Wenzel H. et al., “Theoretical and experimental investigations of the limits to the maximum output power of laser diodes,” New J. Phys. 12(8), 085007 (2010). NJOPFM 1367-2630 10.1088/1367-2630/12/8/085007

[46] Lee T. et al., “Efficient photoacoustic conversion in optical nanomaterials and composites,” Adv. Opt. Mater. 6(24), 1800491 (2018).2195-1071 10.1002/adom.201800491

[47] Shi L. et al., “A fiber optoacoustic emitter with controlled ultrasound frequency for cell membrane sonoporation at submillimeter spatial resolution,” Photoacoustics 20, 100208 (2020). 10.1016/j.pacs.2020.100208 33101926 PMC7569214

[48] Mantri Y. Jokerst J. V., “Engineering plasmonic nanoparticles for enhanced photoacoustic imaging,” ACS Nano 14(8), 9408–9422 (2020). ANCAC3 1936-0851 10.1021/acsnano.0c05215 32806027 PMC8043768

[49] Irisawa K. et al., “Influence of laser pulse width to the photoacoustic temporal waveform and the image resolution with a solid state excitation laser,” Proc. SPIE 8223, 82232W (2012). PSISDG 0277-786X 10.1117/12.907714

[50] Wang L. Zhang C. Wang L. V, “Grueneisen relaxation photoacoustic microscopy,” Phys. Rev. Lett. 113, 174301 (2014). 10.1103/PhysRevLett.113.174301 25379919 PMC4287460

[51] Gao F. et al., “Single laser pulse generates dual photoacoustic signals for differential contrast photoacoustic imaging,” Sci. Rep. 7(1), 626 (2017). SRCEC3 2045-2322 10.1038/s41598-017-00725-4 28377616 PMC5429673

[52] Noimark S. et al., “Polydimethylsiloxane composites for optical ultrasound generation and multimodality imaging,” Adv. Funct. Mater. 28(9), 1704919, (2018). AFMDC6 1616-301X 10.1002/adfm.201704919

[53] Shi Y. et al., “Thermally confined shell coating amplifies the photoacoustic conversion efficiency of nanoprobes,” Nano Res. 9(12), 3644–3655 (2016).1998-0124 10.1007/s12274-016-1234-3

