# Supplementary material for: Plasmon-enhanced optoacoustic transducer with Ecoflex thin film for broadband ultrasound generation using overdriven pulsed laser diode
Source: J Biomed Opt. 2023 Dec 22;28(12):125005. doi: 10.1117/1.JBO.28.12.125005 (PMC10739335; doi:10.1117/1.JBO.28.12.125005)
Supplement: Supplementary file 1 [file JBO_028_125005_SD001.pdf]

Supporting Information

Plasmon-enhanced optoacoustic transducer with Ecoflex thin film for broadband ultrasound generation using overdriven pulsed laser diode

Hamin Na,[a,b] *Jaehyeok Park,*[a,b] *Ki-Hun Jeong*[a,b*]

[a] Department of Bio and Brain Engineering, Korea Advanced Institute of Science and Technology (KAIST), 291 Daehak-ro, Yuseong-gu, Daejeon 34141, Republic of Korea

[b] KAIST Institute for Health Science and Technology (KIHST), Korea Advanced Institute of Science and Technology (KAIST), 291 Daehak-ro, Yuseong-gu, Daejeon 34141, Republic of Korea

*E-mail: kjeong@kaist.ac.kr

	**Ecoflex**	**PDMS**
Coefficient of thermal expansion (CTE) (μm·m^{-1}·℃$^{-1}$)	284.2	266.5
peak-to-peak amplitude of optoacoustic signal (a.u.)	1.64	1.46

Table. S1 Coefficient of thermal expansions (CTE) and peak-to-peak amplitudes of optoacoustic signals from Ecoflex and PDMS-coated NPSs. The high thermal expansion of Ecoflex results in an optoacoustic amplitude increase of 1.12 times compared to the PDMS-coated NPS, which is comparable to a ratio of CTE of Ecoflex to PDMS (~1.07).

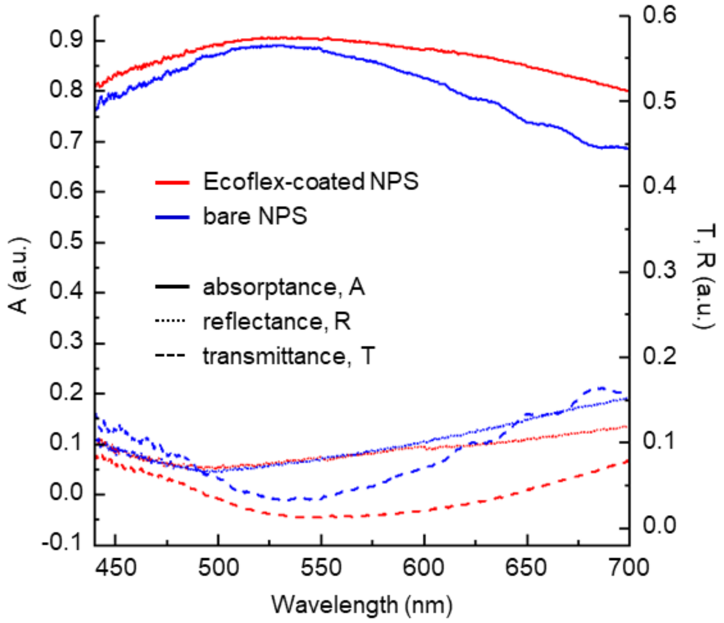

Fig. S1 Optical characteristics including absorptance, reflectance, and transmittance of Ecoflex-coated NPS and bare NPS. The absorptance of Ecoflex thin film is negligible compared to that of NPS.

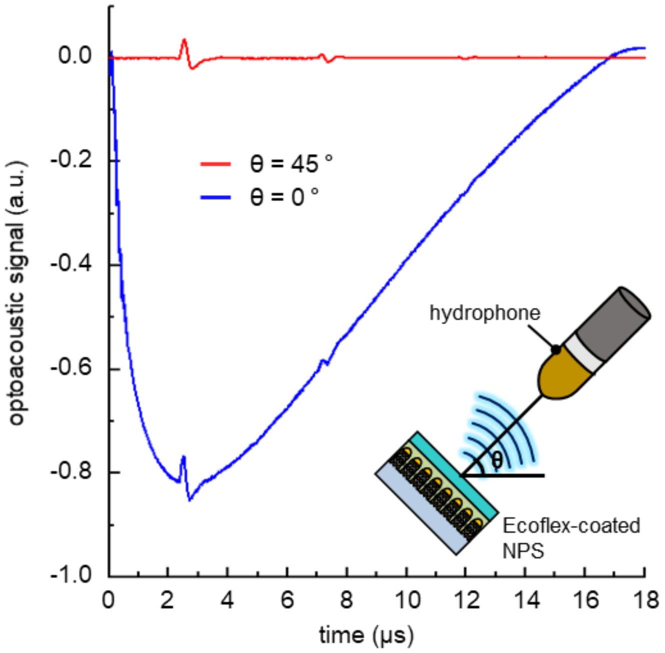

Fig. S2 Optoacoustic signals depending on the angle between the hydrophone and the propagation axis of laser beam. Straight alignment of the laser diode, Ecoflex-coated NPS, and hydrophone directly exposes the hydrophone aperture to the beam path, resulting in baseline distortion due to the high output pulse energy from the overdriven laser diode.

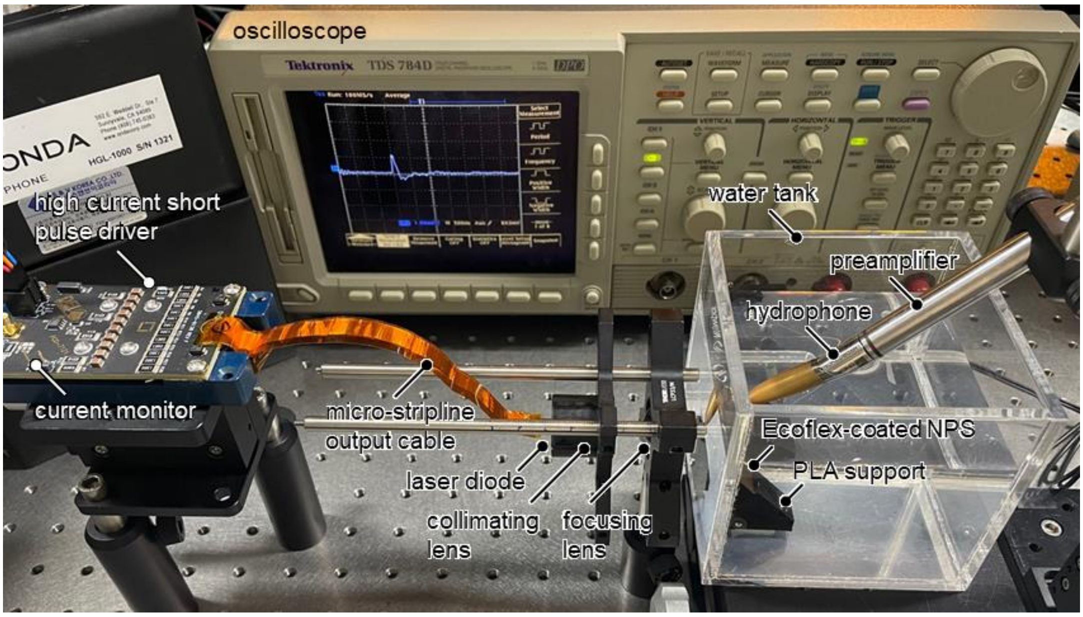

Fig. S3 Optical image of experimental setup for pulsed laser modulation and underwater acoustic measurement. The Ecoflex-coated NPS was positioned at a 45-degree by using a polylactic acid (PLA) support. The hydrophone and preamplifier were connected to a 50 Ω oscilloscope to match the impedances between the preamplifier and the oscilloscope. The voltage signals of acoustic waves were acquired and processed by using LabView interface.

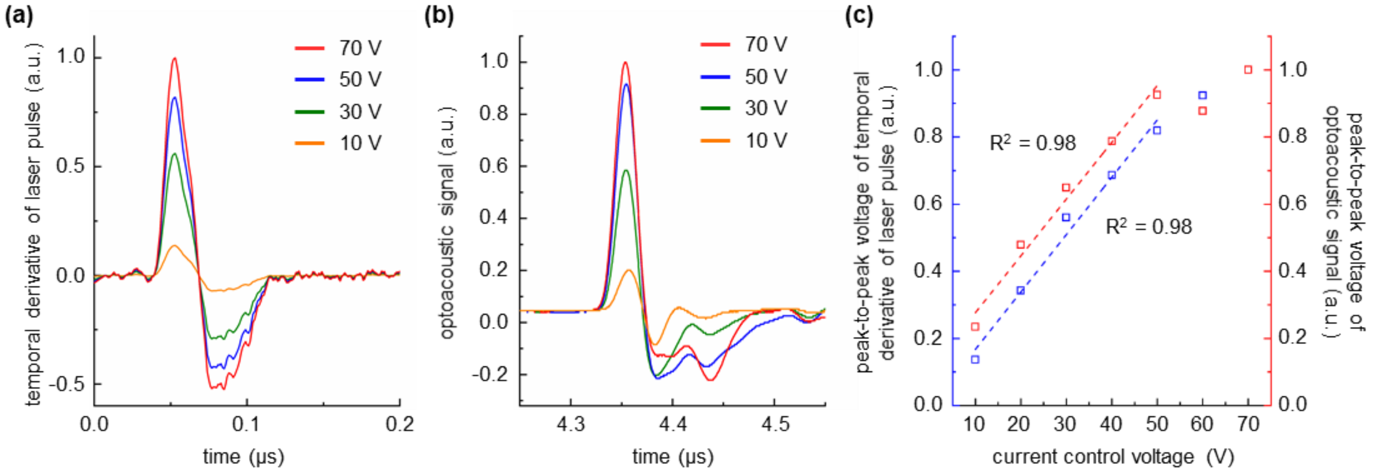

Fig. S4 Plasmon-enhanced optoacoustic signal depending on laser pulse. (a) Temporal derivative of laser pulses induced by different current control voltages from 10 to 70 V. (b) Corresponding optoacoustic signals from Ecoflex-coated NPS. (c) Peak-to-peak voltages of temporal derivative of laser pulses and optoacoustic signals depending on the current control voltage.

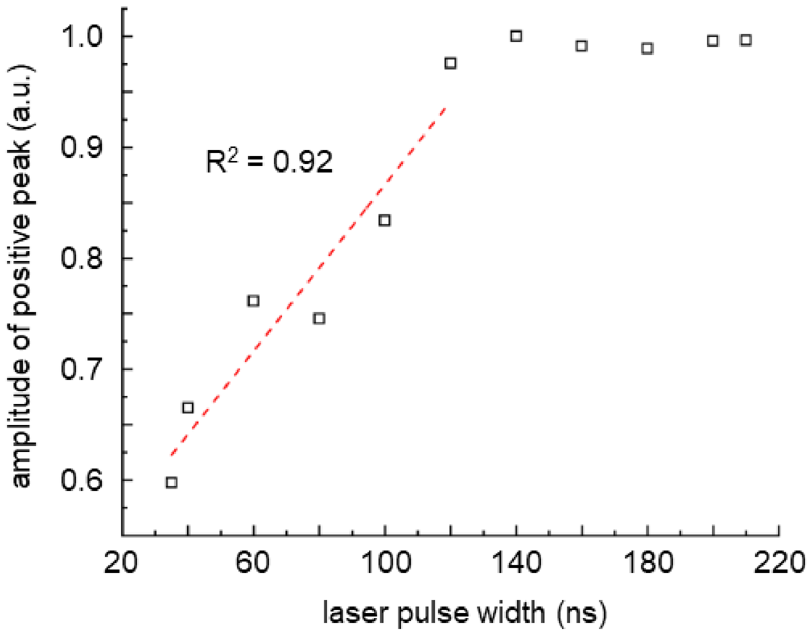

Fig. S5 Optoacoustic amplitude of positive peak corresponding to the compressive wave depending on the laser pulse width. The amplitude of positive peak reaches the maximum amplitude at the pulse width of 120 ns as thermal energy within the Ecoflex thin film saturates.

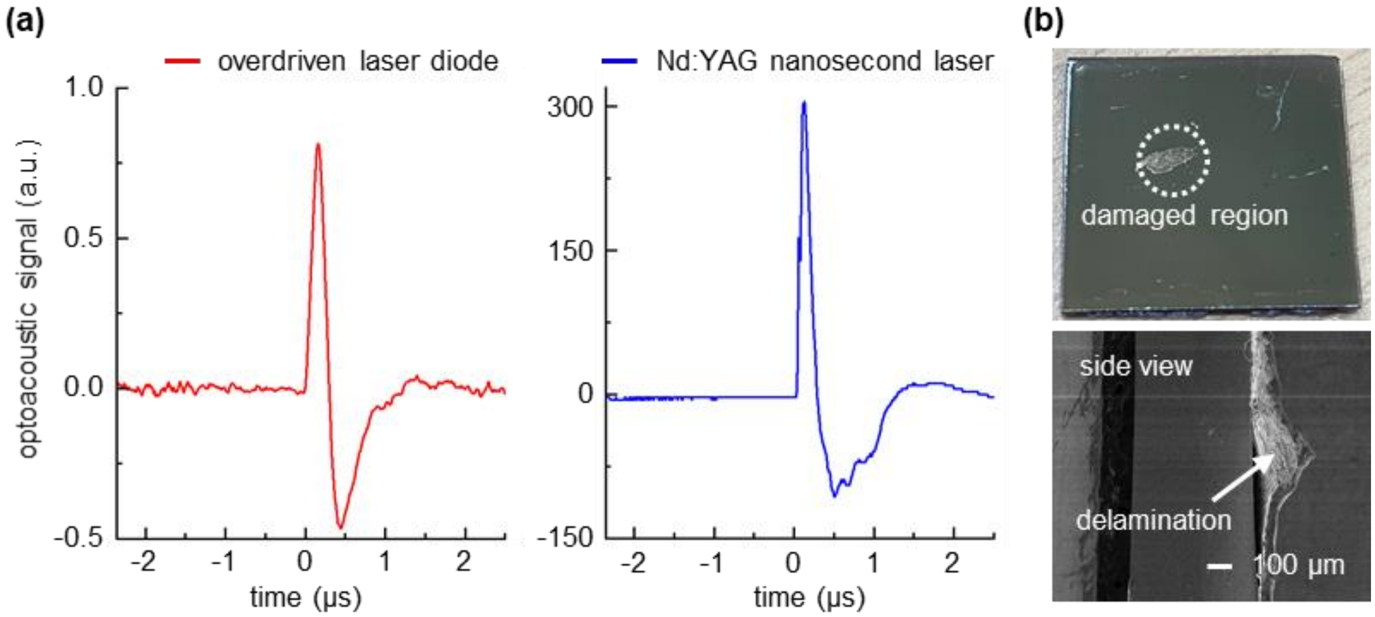

Fig. S6 Plasmon-enhanced optoacoustic generation using an overdriven laser diode and a Nd:YAG nanosecond laser. (a) Optoacoustic signals induced by Ecoflex-coated NPSs with an overdriven laser diode (left) and a Nd:YAG nanosecond laser (right). (b) Optical image of damaged Ecoflex-coated NPS due to the high output pulse energy from Nd:YAG nanosecond laser (top) and cross-sectional scanning electron microscopy (SEM) image of the delaminated Ecoflex thin film (bottom).
